# DIVERSIFYING THE PIPELINE OF GERONTOLOGISTS AT THE UNIVERSITY OF MASSACHUSETTS BOSTON

**DOI:** 10.1093/geroni/igad104.0639

**Published:** 2023-12-21

**Authors:** Edward Miller, Jeffrey E Stokes, Pamela Nadash, Kathrin Boerner, ellen Birchander

**Affiliations:** University of Massachusetts Boston, Boston, Massachusetts, United States; University of Massachusetts Boston, Boston, Massachusetts, United States; University of Massachusetts Boston, Boston, Massachusetts, United States; University of Massachusetts Boston, Boston, Massachusetts, United States; University of Massachusetts Boston, Boston, Massachusetts, United States

## Abstract

The University of Massachusetts (UMass) Boston is a public research university serving approximately 16,000 students. It is the most diverse university in New England and third most diverse university in the nation. UMass Boston prioritizes fostering an anti-racist and health-promoting institutional culture. Consistent with this priority, the Gerontology Department is committed to growing the size and diversity of the professional pipeline in aging services, policy, practice, and research. This presentation describes the multi-pronged approach taken to achieve this objective, while identifying associated facilitators and challenges. Department-wide initiatives include prioritizing work in disparities and a commitment to diversity, equity and inclusion in faculty hiring and implementing curriculum self-assessment and climate survey tools. Doctoral program initiatives include establishing a scholarship in Social Justice and Aging and submitting a training grant application in Health Equity Research. Undergraduate program initiatives include changing the name of the major to “Aging Studies” to make the content clearer and more accessible, proposing a minor in Aging Studies to make it possible for students from across the University to earn a valuable credential, having all courses meet Social and Behavioral requirements in general education (including, in select cases, the Diversity requirement as well), and expanding the number and attractiveness of course offerings (including “Diversity and Aging”). The Department is also implementing an initiative where undergraduates receive paid internships at local-area aging agencies with potential for continued employment post-graduation. Enhancing diversity goes hand-in-hand with growing program enrollment with positive benefits for older adults and their families, caregivers, and communities.

